# Inhibitory effects of tolerogenic probiotics on migratory potential of lupus patient-derived DCs

**DOI:** 10.22038/IJBMS.2021.58438.12982

**Published:** 2021-11

**Authors:** Seyed-Alireza Esmaeili, Ramezan Ali Taheri, Mahmoud Mahmoudi, Amir Abbas Momtazi-Borojeni, Mehdi morshedi, Ali Bahramifar, Mahdi Fasihi-Ramandi

**Affiliations:** 1 Immunology Research Center, Bu-Ali Research Institute, Mashhad University of Medical Sciences, Mashhad, Iran; 2 Immunology Department, Faculty of Medicine, Mashhad University of Medical Sciences, Mashhad, Iran; 3 Nanobiotechnology Research Center, Baqiyatallah University of Medical Sciences, Tehran, Iran; 4 Department of Medical Biotechnology, School of Medicine, Alborz University of Medical Sciences, Karaj, Iran; 5 Trauma Research Center, Baqiyatallah University of Medical Sciences, Tehran, Iran; 6 Molecular Biology Research Center, System Biology and Poisonings Institute, Baqiyatallah University of Medical Sciences, Tehran, Iran

**Keywords:** Chemokine receptor, Lactobacillus Delbrueckii, Lactobacillus Rhamnosus, Systemic lupus – erythematosus, Tolerogenic dendritic cell

## Abstract

**Objective(s):**

The present *in vitro* study aimed to evaluate whether *Lactobacillus delbrueckii* and *Lactobacillus rhamnosus* treatments can induce regulatory phenotype, together with modulating the expression of chemokine receptors (CRs) in dendritic cells (DCs). The CRs of DCs have been found to be involved in the pathogenesis of Systemic lupus erythematosus (SLE) through directing recruitment and migration of immune cells.

**Materials and Methods:**

In brief, monocytes of patients with SLE and healthy donors were isolated and differentiated to regulatory or inflammatory mature DCs through treatment with *L. delbrueckii*, *L. rhamnosus*, mixed probiotics, and LPS.

**Results:**

FACScan analysis showed that the expression of CRs including CXCR3, CCR5, CCR4, and CCR3, was significantly reduced in all probiotic-treated groups of SLE and healthy (control) donors when compared with the LPS treated group.

**Conclusion:**

The results demonstrated that tolerogenic probiotics could prevent or decrease the expression of inflammatory CRs on the surface of tolerogenic DCs during the maturation process.

## Introduction

Systemic Lupus erythematosus (SLE) is a complex progressive inflammatory autoimmune disease, in which various pro-inflammatory immune cells are activated. Several factors, including environmental, genetic, epigenetic, and hormonal factors are involved in the onset of the disease, but the underlying cause of the disease remains unknown. The disease progress is affected by initiation of innate and acquired immune responses and accelerated by repeated excessive immune responses, which ultimately lead to damage in tissues through autoimmune reactions and deposition of immune complexes (1-3). Chronic inflammation and hyperactive immune responses in SLE patients play a determinant role in the pathogenesis of the disease. Several types of immune cells contribute to the priming, incidence, progression, and outcome of the SLE disease. Dendritic cells (DCs), as central innate immune cells, are primary cells that determine the direction of the immune responses to tolerance or inflammatory state in lupus patients (4, 5). These cells are capable of uptalking many antigens and stimulate several immune cell clones, such as autoreactive clones. DCs are necessary for connecting two branches of immune responses and affect acquired immune response outcome, these cells are also responsible for priming and developing SLE. Also, tolerogenic DCs express the low level of co-stimulatory receptors and inflammatory cytokines, which can modulate the regulatory function of other immune cells (6, 7). 

The chemokine receptors, as the immune cell surface receptors, direct the cell recruitment and migration toward primary lymph nodes, thereby inducing inflammatory or regulatory responses. These receptors also mediate the secondary migration of the immune cells to other lymph nodes or non-lymph node organs through chemokine tracing for homing or functional roles. Therefore, down-regulation and/or up-regulation of chemokine receptors can have a direct effect on the outcome of the disease (8, 9). The chemokine receptors, including CXCR3, CCR5, CCR4, and CCR3 are involved in the pathogenesis of the lupus disease. CXCR3 interacts with CXCL9, CXCL10, and CXCL11. CXCR3-expressing immune cells (myeloid DCs, T cells, B cells, plasma cells, and macrophages) exacerbate lupus immunopathogenesis by elevating infiltration of immune cells into inflammatory sites (9, 10) 

Moreover, expression of CCR5 (ligands CCL3, CCL4, and CCL5), CCR4 (ligands CCL17 and CCL22), and CCR3 (ligands eotaxin and eotaxin-2) on different immune cells is associated with the progress of inflammation in SLE (11, 12). CCR5 expression on activated T cells is along with more cell infiltration and inflammation, although it shows a protective effect on lupus when expressed in foxp3^+^ T cells and macrophages that negatively reduce local inflammation and systemic immune responses. Therefore, CCR5 function is cell type-dependent and leads to different responses in various immune cells (13, 14). The increasing expression of CCR4 and its ligands (CCL17 and CCL22) in (MRL-Fas (lpr) lupus mice and patients with lupus nephritis have been shown to be associated with recruitment of T cells to the kidney tissue, which led to reduced number of circulating T cells and progression of SLE disease(14). CCR3 expression is also found to be increased in lupus patients with renal damage and positively resulted in more inflammation (15). CCR4 expression is detected to be positive in both immature DCs (IDCs) and mature DCs (MDCs), but CCR3 and CCR5 expressions are positive in IDCs and down-regulated during maturation (16). CCR3 and CCR5 on IDCs and mDCs are involved in migration and recruitment of blood myeloid DCs (17). Therefore, the aforementioned findings indicate modulation of these chemokine receptors can suppress inflammation by reducing the migratory function of DCs and ameliorate the disease complications and pathogenesis (18, 19). 

Lactobacillus bacteria*,* belonging to the probiotic branch, commonly live in the intestinal tract. These probiotics are able to modulate immune responses and induce tolerance in the intestines (20, 21). *Lactobacillus rhamnosus *and *Lactobacillus delbrueckii* could decrease the expression of co-stimulatory molecules such as CD40, CD86, CD80, and HLA-DR on the surface of DCs, resulting in promotion of tolerogenic phenotypes in DCs during the maturation process (6, 22). These tolerogenic probiotics have anti-inflammatory properties and induce tolerance through reducing inflammation and increasing regulatory mediators by decreasing the number of Th1/Th17 and level of IFN-γ / IL17 as well as increasing Treg frequency and TGF-β level (23, 24). 

 In the present study, we investigated the expression of chemokine receptors, CXCR3, CCR5, CCR4, and CCR3, in the surface of probiotic-maturated tolerogenic DCs (matured by *L. rhamnosus* and *L. delbrueckii*) with monocyte origin to find the effect of these probiotics on migratory lupus-DC patterns. 

## Materials and Methods

***Study design and cell isolation***

Five new SLE patients and five healthy donors were selected and all new SLE patients were enrolled consecutively from the Rheumatology Research Center of the Ghaem Hospital, Mashhad, Iran. All patients according to SLEDAI had at least 4 of the revised SLE classification criteria of the American College of Rheumatology. The protocol of this study was approved by the Human Ethics Committee of Mashhad University of Medical Sciences, and all participants signed a written consent form before entering the study. Patients were excluded from the study if they had received prior treatment. Whole blood sample (10 ml) from healthy individuals and SLE patients (newly diagnosed / not received any medications) was collected in the heparin tubes and rotated for 15 min. After 1 hr incubation, blood samples were diluted with phosphate-buffered saline (PBS) (1/1 ratio) and used for peripheral blood mononuclear cell (PBMCs) isolation by density gradient centrifugation of Ficoll-Hypaque (Cedarlane, Toronto, Canada) according to the standard instructions. To isolate the monocytes, PBMCs were cultured in RPPMI-1640 media ((BioSera, London, UK), for 2 hr at 37 ºC using the plastic adherence method. After isolation of monocytes, FACScan (BD Bioscience, San Diego, CA, USA) evaluated their purity through staining with CD45 FITC/CD14 R-PE (IQProducts, Groningen, the Netherlands), in comparison with FITC/PE-isotype control antibody (mouse IgG1 isotype (IQProducts, Groningen, the Netherlands)). Cells (10^6^) were stained by antibodies and incubated for 1 hr at 4°C, then monocytes were washed three times with PBS and centrifuged at 300 g. Finally, cells were resuspended in 300 μl PBS and analyzed. The data were analyzed using Cell Quest Pro (BD Biosciences, San Jose, CA, USA) or FlowJo 7.6.2 software (Tree Star, Ashland, OR, USA). In addition, morphological shapes of monocytes were confirmed using microscopic 100x and 40x objective lens field.

***Probiotic culture and determine the count of probiotics***

MRS broth (Biolife, Viale Monza, Milano-Italia (LOT: EB4301)) supported the growth of lyophilized* L. rhamnosus* ((ATCC: 9595) (Pasteur Institute of Iran)) and *L. delbrueckii *subsp* lactis* (PTCC: 174) (Iranian Research Organization for Science and Technology (IROST)) in microaerobic conditions at 37 °C for 1 hr. Broth media containing probiotics were then cultured in MRS agar (Biolife, Viale Monza, Milano-Italia (LOT: EB4302)), for 24 hr at 37 °C under microaerobic conditions. The colonies of both probiotics were harvested and the number of each probiotic in 1 ml MRS broth was determined by reverse dilution culture or OD (620 nm) _˟_ 10^8^ formula for next evaluation. 

***Dendritic cell differentiation***

In each well of a 24-well plate, 0.2 x 10^6 ^monocytes/well were cultured in a plate containing fresh complete RPMI supplemented with 100 IU/ml penicillin (Sigma-Aldrich, USA), 100 µg/ml streptomycin (Sigma-Aldrich, USA), and 2 mM L-glutamine (Sigma-Aldrich, USA). To generate immature DCs (IDCs), monocytes were cultured for 5 days and on days of 1 and 3, 10 ng/ml GM-CSFn (RD, Minneapolis, MN, USA) and 10 ng/ml IL-4 (RD, Minneapolis, MN, USA) cytokines were added to culture media, respectively. After 5 days, floated cells were suspended in the media culture and considered as immature DCs (IDCs) based on the appearance and expression of surface markers CD14, CD1a, CD80, CD86, HLA-DR, and CD83 (6). To generate inflammatory and regulatory mature dendritic cells (MDCs), 10 ng/ml GM-CSF and 10 ng/ml IL-4 were added again and incubated for 48 hr in culture with LPS (100 ng/ml), *L.* *delbrueckii*, *L.* *rhamnosus,* and a mix of both probiotics (2 × 10^6^ bacteria/well). All produced DCs were confirmed by flow cytometry.

***Experimental groups***

Mature DC was generated from IDC cells of healthy (control) and SLE patient donors. All IDCs were treated with LPS, *L.Del*, *L.Ram*, and a mix of both probiotics. The cMDCs (control-mature DCs) and pMDCs (patient-mature DCs) were determined as two main groups. cMDC-LPS, cMDC-Del, cMDC-Ram, and cMDC-Mix were considered as subgroups in generated DCs of healthy donors, and pMDC-LPS, pMDC-Del, pMDC-Ram, and pMDC-Mix were considered as subgroups in generated DCs of SLE patient donors. 

***Flow cytometry***

To check the ability of IDCs for antigen uptake, we used the FITC-dextran dye kit (mol. wt 40,000; Sigma) and the flow cytometry method. 5×10^5^ IDCs were incubated with 1 mg/ml FITC-dextran dye for 2 hr at 37 °C, after staining, the cells were washed twice with PBS to remove the free dye. Then, IDCs were adjusted in 200 ul PBS and measured by FACScan (BD, FACS Calibur). Also, the expression of chemokine receptors in both regulatory and inflammatory mature DCs was measured by FACScan. In brief, the Cells were stained by CXCR3-FITC, CCR5-PE, CCR3-FITC, CCR4-PE (R&D system, USA), and FITC/PE-isotype control, and incubated for 45 min at 4 °C and then washed twice with PBS. The cells were adjusted in 200 ul PBS and were measured by FACScan. 

***Data analysis***

All data were analyzed using GraphPad Prism 5 Software (Graph Pad, Inc., San Diego, CA, USA) and SPSS software Ver. 11.5 (IBM Corporation, NY, USA). *P*-values<0.05 were considered statically significant.

## Results

***Measuring the concentration of probiotics in MRS broth***

The number of probiotics in 1 ml MRS broth was calculated through OD absorption at 620 nm, (formula: OD × 8.8× 10^8^) and confirmed by the culture of serial dilution of probiotics. The number of both *L. delbrueckii *and *L. rhamnosus* was determined in the range of 9 ×10^8^ -10^9^ / ml and adjusted in PBS for the next experiments. 

***The results and purity of isolated PBMCs and Monocytes***

In the first step, 1.5 million of PBMCs were isolated from 1 ml of each whole blood heparinized sample, and then PBMCs population was evaluated by flow cytometry which determined that 21% of isolated PBMCs were monocyte. Also, after purification of monocytes (CD45^+^ and CD14^+^ cells), FACScan analysis showed a purity of 85% to 90%. 

***Expression results of immature DC surface markers and IDCs uptake***

**Figure 1:**
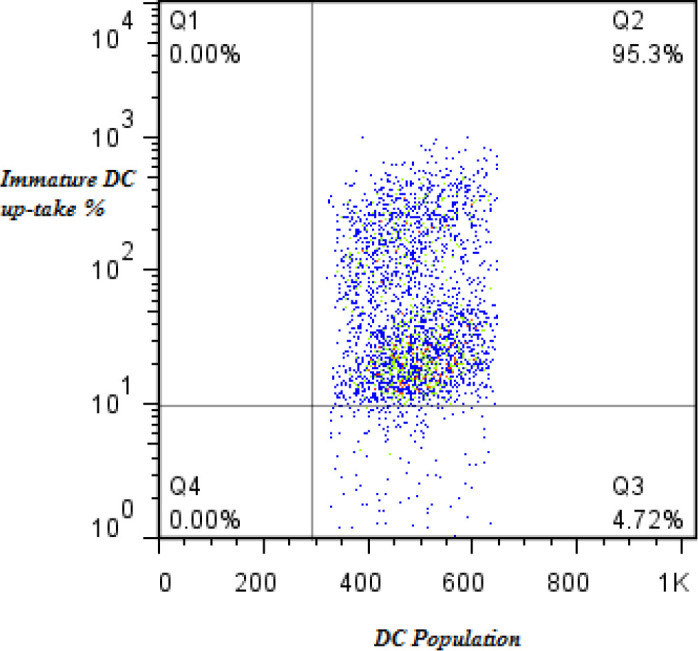
DC uptake assay results. Results of IDCs Ability to uptake FITC-dextran dye. The results showed that IDCs were able to pick up the dye after incubation. Histogram and dot plot of flow cytometry results demonstrated 95.3% and 95.4% of IDCs pick up the dye, respectively

FITC: fluorescein isothiocyanate; IDCs: immature dendritic cells; FL1-H: fluorescence measured in channel 1

IDC was generated during 5 days culture of monocytes in the presence of IL4 and GM-CSF cytokines. The appearance of IDCs under microscopic field, high reduction of CD14, and upexperssion of CD80, CD86, CD1a, CD83, and HLA-DR in compression with monocytes confirmed the correct generation of these cells (6). Before maturation of DCs by *L. delbrueckii*, L*. rhamnosus,* or LPS, we first evaluated the ability of IDCs to uptake FITC-dextran. Our results demonstrated that 95% of generated IDCs could uptake FITC-dextran dye and were proper for following the maturation process (Figure 1).

***Expression results of CXCR3, CCR5, CCR4, and CCR3 in regulatory and inflammatory mature DCs***

**Figure 2:**
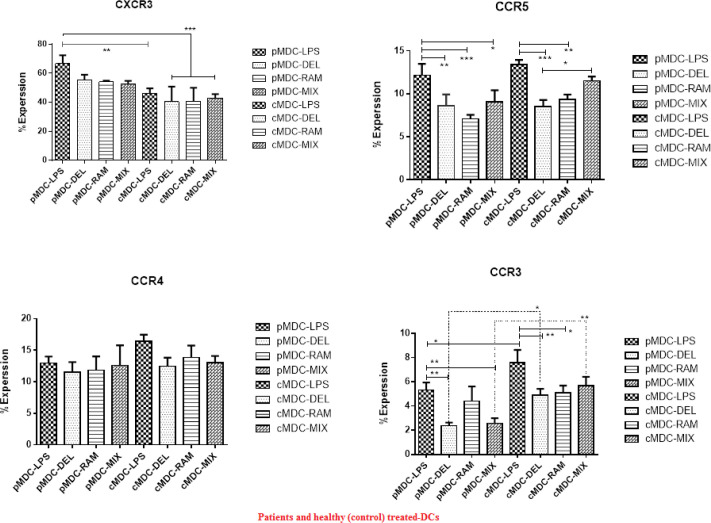
Flow cytometry results. Results of chemokine receptor expression (CXCR3, CCR5, CCR4, and CCR3) in treated MDCs of SLE and healthy donors. (CXCR3): Expression of CXCR3 was decreased in MDC-DEL, MDC-RAM, and MDC-MIX groups of both patients and healthy donors. cMDC-DEL, cMDC-RAM, and cMDC-MIX along with cMDC-LPS have shown lower significant expression compared with pMDC- LPS. (*P*<0.0004). (CCR5): In all probiotics treated groups in both healthy and SLE donors significant reduction was observed compared with LPS groups (*P*<0.0001). (CCR4): In all probiotics treated groups in both healthy and SLE donors reduction was observed compared with LPS groups but not significantly. (CCR3): Expression in probiotics treated groups was significant and lower than LPS treated groups, also lower significance was observed in pMDC-DEL and pMDC-MIX in comparison with corresponding sub-groups of healthy donors (*P*<0.0001)

MDCs were generated during 2 days culture of IDCs in the presence of *L. delbrueckii*, *L. rhamnosus*, mix probiotics, as well as LPS plus IL4 and GM-CSF cytokines. Similar to IDCs CD14 plus CD80, CD86, CD1a, CD83, and HLA-Dr were assessed to confirm the correct maturation process (6). Our results showed that *L. delbrueckii*, *L. rhamnosus,* and mix probiotics with anti-inflammatory properties could decrease significant expression of CXCR3, CCR5, CCR4, and CCR3 in both cMDCs (control-mature DCs) and pMDCs (patient-mature DCs), when compared with LPS-MDC in both healthy and patients groups. CXCR3 expression in all of the probiotic treated groups in comparison with LPS treated groups of healthy and SLE donors was decreased, but a significant reduction in the expression of CXCR3 was observed in cMDC-LPS, cMDC-DEL, cMDC-RAM, and cMDC-MIX in comparison with pMDC-LPS (*P*<0.0004). Similarly, expression of CCR5 in all probiotic treated groups of healthy and SLE donors was significantly decreased. In pMDC groups, pMDC-DEL, pMDC-RAM, and pMDC-MIX showed significant reduction of expression compared with pMDC-LPS (*P*<0.0001). In addition, in control groups (cMDC), a significant reduction was observed in cMDC-DEL and cMDC-RAM in comparison with cMDC-LPS, and between the cMDC-DEL and cMDC-MIX (*P*<0.0001). The results showed that our probiotics could reduce the expression of CCR4 in probiotics-MDC groups in both healthy and SLE donors but this reduction was not significant. Furthermore, expression of CCR4 in pMDC-LPS was lower than in cMDC-LPS. In the case of CCR3 expression, the results showed that matured groups with probiotics in SLE donors had lower expression compared with the LPS group; this reduction was significant in pMDC-DEL and pMDC-MIX (*P*<0.0001). In addition, healthy donors have shown the same results but significant reduction was related to cMDC-DEL and cMDC-RAM. In both SLE and healthy donors, a significant decrease was observed in the corresponding sub-groups and the expression was lower in pMDC-DEL and pMDC-MIX compared with cMDC-DEL and cMDC-MIX (*P*<0.0001) (Figure 2). Also the information of regulatory and inflammatory DCs upon expression of CD14, CD1a, CD80, CD86, HLA-DR, and CD83 surface markers and pattern of inflammatory or regulatory cytokines was considered (6) along with CCR expression to reach better results. 

pMDC: patients matured DC; cMDC: control (healthy) matured DC; MDC: mature dendritic cell; DEL: *Lactobacillus delbrueckii*; RAM: *Lactobacillus rhamnosus*; MIX, both probiotics; SLE: systemic lupus erythematosus. One-way ANOVA test was used to statically analyze. *(*P*≤0.05), ** (*P*≤0.005), and *** (*P*≤0.001)

## Discussion

The role of *L. delbrueckii* and *L. rhamnosus *has been proven in various studies(23, 25). In a previous *in vitro* study, we entirely evaluated the regulatory effect of *L. delbrueckii* and *L. rhamnosus* probiotics to generate tolerogenic DCs, with reduced inflammatory surface markers and cytokines in both healthy and SLE donors (6). Also, we confirmed the regulatory role of the mentioned probiotics in the SLE animal model study and have shown after six-month daily oral gavage the number and function of inflammatory cells decreased. CRs are the main receptors in homing, migration, and remigration of DCs, and DCs are determinative cells for control or progression of inflammation in lupus. In a previous *in vivo* study, we found the migration and inflammation rate were decreased in mice renal biopsy tissues that probably contributed to the role of chemokine receptors in the migration manner of inflammatory cells. Therefore, like a previous *in vitro *and* in vivo* study, we decided to evaluate the effect of *L. delbrueckii* and *L. rhamnosus *probiotics on the expression of chemokine receptors on maturated DCs.

 The results of the present study showed that according to the prediction, probiotics could significantly decrease expression of CCR3, CCR4, CCR5 and CXCR3 on the surface of generated DCs in both healthy and SLE cell populations. For this purpose, we first produced IDCs and then evaluated their ability to uptake antigens. We showed that IDCs differentiated to regulatory and inflammatory mature DCs in culture with probiotics and LPS, respectively, which was confirmed by expression of CD14, CD1a, CD80, CD86, HLA-DR, and CD83 surface markers and pattern of inflammatory or regulatory cytokines (6). Our probiotics could generate mature DCs with tolerogenic properties leading to reduced expression of all chemokine receptors in comparison with MDC-LPS in both healthy and SLE donors(6). In the case of chemokine receptors, our present results showed that both *L. delbrueckii* and *L. rhamnosus* probiotics separately and in combination could decrease expression of CXCR3, CCR5, CCR4, and CCR3 on the surface of generated DCs. These chemokine receptors are involved in the migration of DC to different inflammation sites (17, 26, 27) and, therefore, their reduction can prevent DC migration and whereby control inflammation. We showed a reduction in the expression of chemokine receptors occurred in both SLE and healthy donors, but this reduction was more in SLE patients. In SLE and healthy donors, CXCR3 results demonstrated that probiotic treated groups had a lower expression compared with the LPS treated group, in which the reduced expression can be due to the tolerogenic effect of used probiotics during the maturation process of DC and shifting to regulatory properties, including low expression of surface markers including CXCR3. In some studies, the role of *L. delbrueckii* and *L. rhamnosus* in the reduction of surface markers of DCs have been proven (6, 24). Due to the role of CXCR3 in recruitment and infiltration of Th1 and Th17 and induction of renal injury (28), it is possible that the studied probiotics, through reduction of CXCR3, inhibit migration of DCs to inflammation site and reduce injury in lupus nephritis. 

Regarding expression of chemokine receptors CCR5, CCR4, and CCR3 in all probiotic treated groups of healthy and SLE donors, we observed a reduction in the expression of CCRs in comparison with LPS treated group. The patterns of reduction in expression of CCR5 and CCR4 were similar but the expression reduction of CCR3 was more, and in SLE patients expression of the CCR3 marker was significantly suppressed. Since these reductions occurred in tolerogenic DCs compared with the inflammatory DC cells, concurrent with the reduction of other surface inflammatory markers, the chemokine receptors have also been reduced by the anti-inflammatory effect of probiotics. It is further supported by the other studies showing the anti-inflammatory ability of these probiotics(29). CCR5, CCR4, and CCR3 are the main causes of immune cell migration and exacerbation of inflammation(30, 31). Thus, *L. delbrueckii* and *L. rhamnosus* can decrease these markers and as a strategy can be used as a daily complimentary to treatment of the patients who suffer from increased inflammation and tissue injury. 

## Conclusion

Probiotics *L. delbrueckii* and *L. rhamnosus* could decrease expression of CXCR3, CCR5, CCR4, and CCR3 on the tolerogenic phenotype of DCs in healthy and SLE donors. This reduction indirectly can reduce inflammation through blockage of cell migration to inflammatory sites. In SLE patients, because of the repeated inflammation and existence of inflammatory pattern of cells, the reduction was more. Considering the characteristics of probiotics such as safety, price, daily ease of use, prophylactic and therapeutic roles, the potential for use with other drugs, and the possibility of improving the flora in the intestines, it is suggested that these probiotics be considered as an effective approach to ameliorate SLE symptoms in clinical studies or used as a complementary drug. 

## Authors’ Contributions

MFR conceived and designed the study. SAE and RAT conducted the experimental work and collected the data and analyzed data. MM, and AB drafted the manuscript. AAM and MM evaluated the data and critically revised the manuscript. All authors approved the final version of the manuscript.

## Funding

This study has been financially supported by Mashhad University of Medical Sciences (grant number: 930895)

## Conflicts of Interest

The authors declare that they have no conflicts of interest.
